# Staphyloxanthin as a Potential Novel Target for Deciphering Promising Anti-*Staphylococcus aureus* Agents

**DOI:** 10.3390/antibiotics11030298

**Published:** 2022-02-23

**Authors:** Rana A. Elmesseri, Sarra E. Saleh, Heba M. Elsherif, Ibrahim S. Yahia, Khaled M. Aboshanab

**Affiliations:** 1 Department of Microbiology, Faculty of Pharmacy, Misr International University (MIU), Cairo 19648, Egypt; rana.elmesseri@miuegypt.edu.eg (R.A.E.); heba.magdy@miuegypt.edu.eg (H.M.E.); 2 Department of Microbiology & Immunology, Faculty of Pharmacy, Ain Shams University (ASU), Cairo 11566, Egypt; sarradeif@pharma.asu.edu.eg; 3 Laboratory of Nano-Smart Materials for Science and Technology (LNSMST), Department of Physics, Faculty of Science, King Khalid University, P.O. Box 9004, Abha 61441, Saudi Arabia; ihussein@kku.edu.sa; 4 Research Center for Advanced Materials Science (RCAMS), King Khalid University, P.O. Box 9004, Abha 61413, Saudi Arabia; 5 Nanoscience Laboratory for Environmental and Biomedical Applications (NLEBA), Semiconductor Laboratory, Department of Physics, Faculty of Education, Ain Shams University, Roxy, Cairo 11757, Egypt

**Keywords:** anti-virulence, staphyloxanthin, *Staphylococcus aureus*, CrtM, CrtN, MRSA

## Abstract

*Staphylococcus aureus* is a fatal Gram-positive pathogen threatening numerous cases of hospital-admitted patients worldwide. The emerging resistance of the pathogen to several antimicrobial agents has pressurized research to propose new strategies for combating antimicrobial resistance. Novel strategies include targeting the virulence factors of *S. aureus*. One of the most prominent virulence factors of *S. aureus* is its eponymous antioxidant pigment staphyloxanthin (STX), which is an auspicious target for anti-virulence therapy. This review provides an updated outline on STX and multiple strategies to attenuate this virulence factor. The approaches discussed in this article focus on bioprospective and chemically synthesized inhibitors of STX, inter-species communication and genetic manipulation. Various inhibitor molecules were found to exhibit appreciable inhibitory effect against STX and hence would be able to serve as potential anti-virulence agents for clinical use.

## 1. Introduction

*Staphylococcus aureus* (*S. aureus*) is one of the most frequent and virulent human pathogens. It is one of the leading causes of community-acquired and hospital-acquired infections [1]. Clinical manifestations caused by *S. aureus* include respiratory tract infections, skin infections, toxic shock syndrome, sepsis, osteomyelitis, meningo-encephalitis and bacterial endocarditis [2,3]. *Staphylococcus aureus* poses a serious public threat, in particular the multidrug-resistant strain termed as methicillin-resistant *S. aureus* (MRSA), which has raised a red flag as a global health concern. In 2019, MRSA was listed as a priority on top of the antibiotic-resistance threat report published by Centers for Disease Control and Prevention (CDC) [4].

Anti-virulence therapy is expected to provide a useful alternative to combat the horrific resistance against conventional antibiotic therapy. Virulence factors are essentially pathogenic products that assist the pathogen evade the host immune system [5,6].

Recently, virulence factors have been considered novel viable targets for research to get past both of the emergence of drug resistance, as well as the scarcity of novel antibiotics [7]. Previous studies have revealed very promising results of anti-virulence therapy including targeting biofilm production, toxin production, bacterial adhesion and key enzymes [8,9,10].

Based upon the urgent need for effective new anti-pathogenic treatments against MRSA, this review focuses on the carotenoid pigment of *S. aureus*. Staphyloxanthin (STX) is one of the main virulence factors present in more than 90% of *S. aureus* isolates [11]. This crucial virulence factor can help *S. aureus* elude the host’s innate immune defense. The most prominent structural feature of carotenoids is the abundant delocalization of the polyene π-electrons that enables them to absorb visible light, conferring them intense colors that vary from yellow to red [12].

The yellow carotenoid pigment is well known for its ability to act as an antioxidant, having multiple conjugated double-bonds that facilitate the detoxification of reactive oxygen species generated by the host immune system [13]. Earlier works concluded that STX pigment can protect *S. aureus* against oxidative stress by neutrophils. After the engulfment of *S. aureus* by neutrophils, hydrogen peroxide is converted to hypochlorous acids by the aid of myeloperoxidase. The capability of *S. aureus* to evade oxidative stress by hypochlorous acid and reactive chlorines is due to their ability to defuse myeloperoxidase. Furthermore, STX has the ability to scavenge free radicals, singlet oxygen, hydrogen peroxide and hypochlorous acid. The mechanism by which STX acts has not yet been determined, but its activity is presumed to be a result of a highly conjugated isoprenyl tail reacting with oxidants [6,14].

Staphyloxanthin virulence is evidently correlated to its antioxidant activity, since pigmented strains of *S. aureus* were more resistant to killing by neutrophils than non-pigmented strains in mouse models [15]. The biosynthetic pathway of STX by *S. aureus* starts with dehydrosqualene synthase (CrtM) that catalyzes the condensation of two farnesyl diphosphates to produce presqualene diphosphate [16]. 4,4′-Diapophytoene synthase (CrtM), 4,4′-diapophytoene desaturase (CrtN), 4,4′-diaponeurosporene oxidase (CrtP), glycosyltransferase (CrtQ), and acyltransferase (CrtO) are five enzymes famous for governing the biosynthetic pathway of STX by *S. aureus* [17]. In a mouse model, a CrtM-deficient *S. aureus* mutant failed to evade the host neutrophil-based killing [18]. Hence, the findings of the previous study suggest the possibility of inhibiting the biosynthetic pathway of STX by various inhibitor molecules.

**Figure 1:**
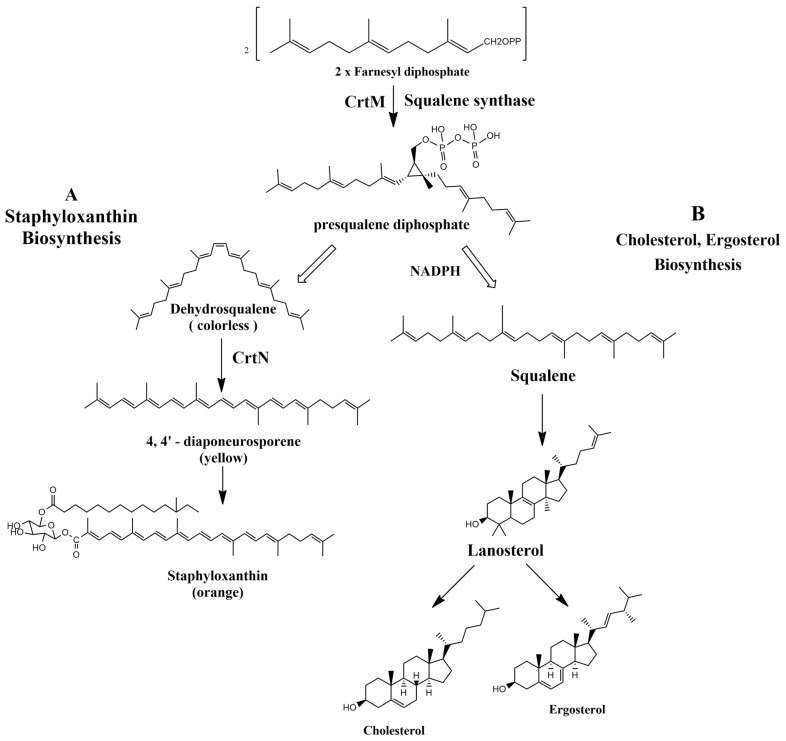
(**A**) Staphyloxanthin biosynthesis pathway in *S. aureus*. (**B**) Cholesterol and ergosterol biosynthesis pathway.

Controlling the biosynthetic pathway of the carotenoid pigment would be quite advantageous since it would limit the survival of *S. aureus* infections in humans and render the pathogen fragile against the host immune response. Both CrtM and CrtN confer novel targets for anti-virulence therapy since they are responsible for the production of *S. aureus* yellow carotenoid pigment (Figure 1) [10,19]. On grounds of this concept, the overall aim of this review is to suggest potential STX inhibitor candidates that are either naturally present or chemically synthesized with potent anti-virulence activity against *S. aureus* strains.

## 2. STX Inhibitors

Several staphyloxanthin (STX) inhibitors have been proved in literature as displayed in Table 1. The chemical structures and structure activity relationship (SAR) of various STX inhibitors are presented in Table 1 and Table 2. The source and mechanism of action as well as the biological activities of the respective inhibitors are discussed as follows.

### 2.1. Naturally Occurring STX Inhibitors

#### 2.1.1. Flavonoids

Flavone, found in many plants and the mainstay of all flavonoids, is well known to have a wide array of biological activities, such as potent antioxidants, antifbacterial, antifugal as well as anticancer activities. Minimal concentrations, as low as 50 μg/mL, of flavone were found to significantly inhibit the yellow carotenoid pigment in *S. aureus*. Antibiotic-resistant *S. aureus* were markedly affected in the presence of flavone which offered a remarkable vulnerability of the strains to hydrogen peroxide. Flavone has also contributed to the inhibition of α-hemolysin and downregulated α-hemolysin gene (hla), resulting in demolishing the hemolysis of red blood cells by *S. aureus* [20]. In 2017, myricetin, a flavonoid commonly present in fruits and vegetables, was reported to inhibit multiple virulence factors in *S. aureus*. Among the most prominently inhibited virulence factors were STX pigment production, biofilm formation, adhesion to target cells and red blood cells hemolysis. Gene expression analysis revealed a downregulation of *saeR* regulator. In vivo, myricetin could efficiently demolish *S. aureus* infection in a larvae model of *Galleria mellonella* [21]. Although most flavonoids are considered safe, there have been reports of toxic flavonoid–drug interactions, liver failure, contact dermatitis and hemolytic anemia [22].

#### 2.1.2. Rhodomyrtone

**Figure 2:**
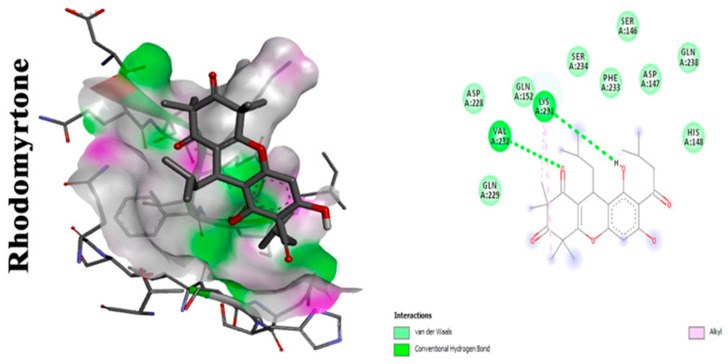
Molecular docking analysis showing (two-dimensional) 2D and (three-dimensional) 3D representation of interaction patterns of rhodomyrtone with CrtM receptor [25].

A study conducted by Leejae et al. revealed rhodomyrtone, a compound that naturally exists in *Rhodomyrtus tomentosa* leaves. It is a potent antibacterial agent against many Gram-positive bacteria. It has the advantage of being nontoxic, as its MIC against most of the Gram-positive bacteria is 0.5–1 µg/mL when tested in humans [23]. *Rhodomyrtone* showed promising results when used on *S. aureus* infected blood specimens in ex vivo assays. Several concentrations of the phytocompound (0.25×, 0.5×, 1×, 2×, and 4× minimum inhibitory concentration (MIC)) were tested on *S. aureus* isolates. The rhodomyrtone-treated cells were defenseless against the action of innate immunity. A comparative study was held to differentiate between the extent of pigment production in treated and untreated cells with rhodomyrtone. The latter was less prone to oxidative radicals generated by human innate immunity. The action of rhodomyrtone is depicted to be due to its ability to reduce pigment production, leading to an increased susceptibility of the cells to toxic free radicals. Additional effects of rhodomyrtone on bacterial virulence include its effect on the synthesis of DnaK protein, responsible for numerous biological activities of the cells, and/or Sigma factor σB where it has led to the downregulation of this gene resulting in a notable susceptibility of the treated cells to oxidative stress [24]. Molecular docking analysis showing 2D (on the right panel) and 3D (on the left panel) representation of interaction patterns of rhodomyrtone with dehydrosqualene synthase receptor is displayed in Figure 2.

#### 2.1.3. Marine Bioresource: Chitosan

Chitosan is a sugar that is naturally present in the outermost shells of *Portunus sanguinolentus*. It was found to exhibit several activities such as antibacterial, antifungal and antioxidant activities as well as some anti-virulent characteristics against MRSA. Staphyloxanthin inhibition was among its principal anti-virulent properties when the extracted (100 µg/mL) and commercial (200 µg/mL) forms of chitosan were tested on MRSA strains at their biofilm inhibitory concentrations (BIC). Moreover, the extracted chitosan was found to have a dose-dependent antibiofilm efficacy against various MRSA strains, as well as proved efficacy in disrupting the thick exopolysaccharide (EPS) layer [26]. Additionally, chitosan is generally safe and did not have any cytotoxic effects when tested on lung epithelial cell lines [27].

#### 2.1.4. *Pogostemon heyneanus* and *Cinnamomum tamala* Essential Oils

Essential oils (EOs) extracted from aromatic plants, such as *Pogostemon heyneanus* and *Cinnamomum tamala*, have been tested against MRSA isolates. The EOs were found to exhibit an inhibitory effect on STX production and hemolysin activity at their minimum biofilm inhibitory concentration (MBIC). The essential oils of *P. heyneanus* and *C. tamala* showed MIC at the range of (2–6% *v*/*v*). The effect of EOs and nerolidol to disrupt the biofilms of MRSA strains was determined at its sub MIC (1/2 and 1/4). *P. heyneanus* EO showed percentage inhibition of biofilms in the range of 60–80% at concentrations ranging from 3 to 0.5% *v/v* whereas *C. tamala* EO showed reduction in biofilms in the range of 55–65% at sub MIC (1–3 *v*/*v*). Molecular docking studies were held using CrtM enzyme, the lead enzyme of STX biosynthesis, and the major compounds of EOs. The results revealed that one of the components of EOs, (E)-nerolidol, had higher binding affinity (−6.65 kcal mol^−1^) to CrtM than other involved compounds [28]. A previous study was conducted to evaluate the effectiveness of a mixture of Curcuma longa, Zanthoxylum limonella and Pogostemon heyneanus essential oils in 1:1:2 ratio at 5%, 10% and 20% concentration against blackflies and its dermal toxicity using rat models. The results revealed that the tested repellant was safe and no significant clinical and behavioral changes were detected [29].

#### 2.1.5. 2-Hydroxy-4-Methoxybenzaldehyde (HMB)

Kannappan et al. conducted a study using 2-hydroxy-4-methoxybenzaldehyde (HMB), which was found to confer anti-virulence effects against the notorious MRSA. The MIC of HMB was detected to be 1024 μg/mL. The compound possessed a notable STX inhibition when tested on MRSA at a sub-inhibitory concentration (200 μg/mL). Real time PCR (qPCR) analysis revealed that HMB treatment selectively attenuates stress regulatory genes, such as *saeS* and *SigB* in MRSA isolates; the selective effect against those genes results in significant inhibition of the virulence array, such as lipase, hemolysin, and nuclease in MRSA. Furthermore, in vitro studies supported the non-toxic influence of HMB on peripheral blood mononuclear cells (PBMC). As a result, HMB is recognized as a propitious anti-virulence candidate against MRSA [30]. Moreover, it was found to have antibacterial, antifungal, antipyretic and antioxidant properties [31]. A previous study was carried out on the safety profile of HMB to determine the possibility of being added in and on food as a new flavoring agent. The results of the study revealed that HMB did not have any safety concern when used in the intended levels [32].

#### 2.1.6. Myrtenol

Myrtenol is a well-known phytocompound famous for its agreeable aroma and traditionally known for treating inflammation, anxiety and gastrointestinal pain. It is also considered as a promising herbal product for treatment of allergic asthma [33]. It was shown to be successful at inhibiting STX production and biofilm formation in MRSA-treated cells. Its anti-virulence activity has been reported to be concentration-dependent, between 75, 150, and 300 μg ml^–1^, without any cytotoxic effect on PBMC. The inhibition of STX production led to increasing the susceptibility of MRSA cells to oxidative radicals as well as human whole blood killing. Downregulation of the global regulator sarA and its mediated virulence genes was revealed upon MRSA cell treatment with myrtenol [34].

#### 2.1.7. *Euphorbia tirucalli* Latex

Antipathogenic properties of *Euphorbia tirucalli* latex have been evaluated in vitro and in vivo. The latex did not have an inhibitory effect on the growth of *S. aureus.* Nonetheless, the latex of *Euphorbia tirucalli* (LET) showed a remarkable STX reduction ability. In vivo, a LET dose of 10 μL/kg could aid the survival of *Tenebrio molitor* larvae previously infected with a lethal dose of *S. aureus.* Hence, LET could set a propitious example of a successful antimicrobial against *S. aureus* [35]. It also has various medicinal uses such as in cases of cough, asthma, warts and rheumatism. The sap of the *Euphorbia tirucalli* was proved to be irritating to the human eyes and may result in kerato-conjunctivitis [36].

#### 2.1.8. *Schinus terebinthifolia* Leaf Lectin

In 2019, Lima et al. studied the antimicrobial activity of the lectin derived from the leaves of *Schinus terebinthifolia* (SteLL) againt *S. aureus* cells. SteLL treatment succeeded in decreasing STX production in *S. aureus* using sub-inhibitory concentrations (0.0065× MIC, 0.125× MIC, 0.25× MIC and 0.5× MIC). Furthermore, SteLL synergized ciprofloxacin activity against *S. aureus* and hence SteLL has been recommended as a novel candidate for anti-virulence therapy [37]. Moreover, the result of Ramos et al. 2019 revealed the antitumor activity of SteLL against sarcoma 180. However, hepatic and renal toxicity was observed in animal models [38].

#### 2.1.9. *Callistemon citrinus* Skeels

*Callistemon citrinus* Skeels and its isolated compounds are famous for their recognizable pharmaceutical uses such as in rheumatism, diarrhea and dysentery [39]. Its antimicrobial activity has been tested on MRSA as well as methicillin-sensitive *S. aureus* (MSSA) cells to study their anti-virulence abilities. Pulverulentone A (C1), one of the most biologically active isolates of the methylene chloride-methanol extract (MME) of *C. citrinus*, displayed potent inhibition of STX biosynthesis at half MIC (62.5 μg/mL) in MRSA and MSSA by 55.6% and 54.5%, respectively. It also revealed significant inhibition of biofilm formation at half MIC for up to 71% in MRSA and 62.3% in MSSA [40]. 

#### 2.1.10. The Essential Oil of *Eugenia brejoensis* L. (Myrtaceae)

The essential oil of *Eugenia brejoensis* L. (Myrtaceae) (EbEO) has been studied in Brazil due its antimicrobial activities. It has been found to have a moderate larvicidal activity against the yellow fever mosquito *Aedes aegypti* [41]. The inhibitory profile of EbEO was scrutinized by Filho et al. against various *S. aureus* isolates including multidrug-resistant (MDR) strains. The essential oil was capable of inhibiting the growth of *S. aureus* cells at concentrations ranging from 8–516 μg/mL. Using sub-inhibitory concentrations of EbEO on *S. aureus*, a significant reduction in the production of the carotenoid pigment was observed that led to an impaired defense against oxidative radicals. Furthermore, it reduced the hemolytic activity of *S. aureus*, thus interfering with its ability to survive in human blood. In vivo studies on *Caenorhabditis elegans* and *G. mellonella* infected with *S. aureus* further confirmed the ability of EbEo treatment to markedly decrease the bacterial load and infection severity in the models [42].

#### 2.1.11. *Ginkgo biloba* Exocarp Extract

*Ginkgo biloba* L. exocarp extract (GBEE) is known for having antiproliferative activity on cancer cell lines, with no observable adverse reactions when used in clinical practice [43]. Moreover, it is a promising safe approach for treating different neurological disorders such as Alzheimer’s disease, stroke and traumatic brain injury [44]. Recent studies have disclosed GBEE’s ability to possess antibacterial effects. Wang et al. examined the inhibitory activities of GBEE on MRSA virulence. The study pointed out that GBEE could inhibit *S. aureus* and MRSA biofilm synthesis in a concentration-dependent manner. GBEE could also destroy preformed biofilm of the strains at a concentration of 12 μg/mL. Quantitative transcriptional analysis unveiled the downregulation of virulence genes related to resistance as the global regulator gene *SigB* after 12 h of GBEE treatment, and notable downregulation of *icaA* and *sarA* after 6 h of treatment. Furthermore, GBEE resulted in the downregulation of *hld* gene along with evident STX inhibition [45].

#### 2.1.12. Carvacrol

**Figure 3:**
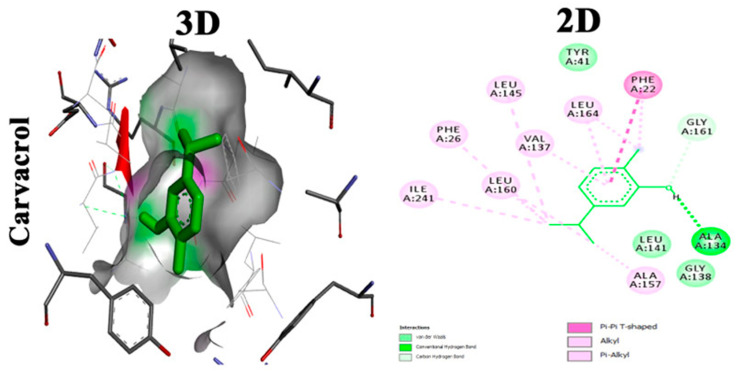
Molecular docking analysis showing 2D and 3D representation of interaction patterns of carvacrol with CrtM receptor [25].

Carvacrol is a phyto-deravative essential oil well known for its aromatic properties that contribute to its wide use as a spice. It has a wide range of biological activities such as anti-inflammatory, analgesic, antipyretic and antimicrobial activities [46]. Moreover, it was found to be clinically safe and tolerable when given to healthy individuals [47]. Selvaraj et al. studied the anti-virulence capabilities of carvacrol against MRSA. Sub-inhibitory concentrations of carvacrol (25, 50, and 75 μg/mL) have been tested against numerous MRSA isolates to detect antibiofilm activity. Carvacrol inhibited the production of STX that rendered MRSA isolates susceptible to reactive oxygen species and whole blood killing. Transcriptomic analysis using (qPCR) revealed the downregulation of sarA gene alongside the downregulation of CrtM gene in the treated isolates. Computational molecular docking unveiled a high affinity of carvacrol towards CrtM gene and SarA regulator in *S. aureus*. In vivo studies hypothesized the capability of carvacrol to mitigate the propagation of MRSA infection in the model as well as maintain a nontoxic effect on the cells of *G. mellonella* larvae [25]. Molecular docking analysis showing two-dimensional (2D) and three-dimensional (3D) representation of interaction patterns of carvacrol with CrtM receptor is displayed in Figure 3.

#### 2.1.13. Thymol

**Figure 4:**
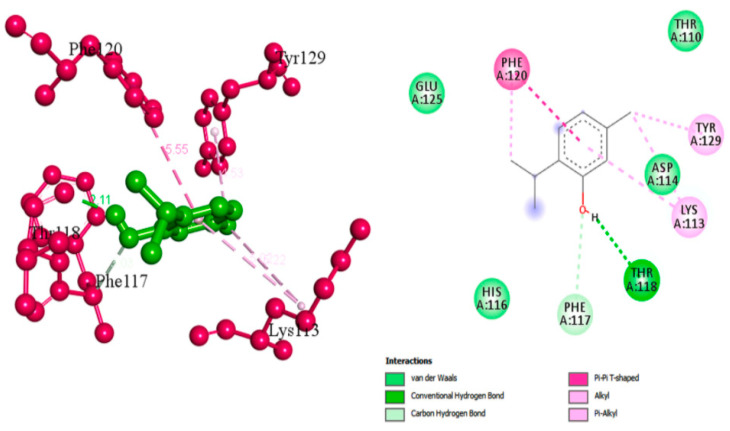
Molecular docking analysis showing 2D (on the right panel) and 3D (on the left panel) representation of interaction patterns of thymol with dehydrosqualene synthase receptor [49].

Thymol has a wide range of therapeutic actions including malignant, cardiovascular and metabolic disorders [48]. Valliammai et al. evaluated the inhibitory activity of thymol against the golden-yellow pigment of MRSA strains. Multiple concentrations of thymol (25, 50 and 100 µg/mL) were used to treat MRSA isolates. Dose-dependent STX inhibitory activity of thymol treatment was observed when compared to positive controls with 90% STX inhibition at 100 µg/mL. Growth curve and cytotoxicity analyses revealed the non-killing nature of thymol at the concentration of 100 µg/mL on MRSA cells and its non-cytotoxic effect on PBMC, respectively. In silico and spectrometric analyses suggested that thymol anti-STX activity could be contributed to by its binding affinity to CrtM receptor in MRSA (Figure 4). Furthermore, oxygen susceptibility assays and ex vivo blood survival assay revealed the compromised ability of the treated MRSA cells to resist oxidative radicals. In addition, STX biosynthesis inhibition resulted in enhancing membrane fluidity and sensitized the treated cells to polymyxin B antibiotic [49].

#### 2.1.14. Hesperidin

**Figure 5:**
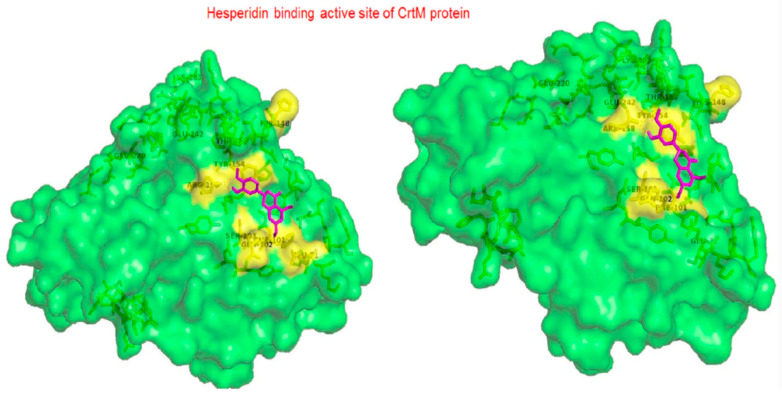
Molecular docking analysis showing 3D representation of interaction patterns of hesperidin with dehydrosqualene synthase receptor.

Hesperidin is well known in the treatment of type II diabetes and cardiovascular disorders, and has potent anti-inflammatory and antioxidant properties. It is generally safe with no adverse effects when tested on humans and animal models [50]. A recent study held by Vijayakumar et al. evaluated the potential anti-infective effect of hesperidin, a flavanone glycoside of plant origin, against MRSA clinical isolates. Hesperidin drastically reduced the production of the carotenoid pigment of MRSA isolates which in turn compromised the survival of MRSA under oxidative stress imposed by H_2_O_2_. Staphyloxanthin inhibition is believed to be due to the downregulation of *crtM* gene in MRSA cells. Computational analysis using molecular docking confirmed the capability of hesperidin binding to CrtM proteins involved in STX production (Figure 5). Moreover, hesperidin was proved to have other anti-virulence activities against clinical isolates of *S. aureus*, such as the significant inhibition of hemolysin, autolysin, and lipase, as well as its antibiofilm potential [51].

### 2.2. Chemically Synthesized Inhibitors

#### 2.2.1. Indole and Halogenated Indoles

Indole acts as an efficient probe that may help in the development of new drugs treating challenging diseases such as lung cancer. Several studies have emphasized the efficacy of indole derivatives such as indole-3-carbinol and indole-3-carboxaldeh as anti-cancer agents [52]. Probing into the effect of indole and its derivatives, one study carried out in vitro and in vivo studies to examine the inhibitory effect of indole and its derivative 7-benzyloxyindole (7BOI) on *S. aureus* in relevance to STX production and hemolytic activity. *S. aureus* was more prone to whole blood killing and oxidative stress when treated with indole or its derivative (7BOI). An in vivo model of nematode *Caenorhabditis elegans* infected with *S. aureus* and treated with (7BOI) proved successful diminishing of virulence in *S. aureus*. Real-time qRT-PCR analysis confirmed the downregulation of virulence genes α-hemolysin gene (*hla*), enterotoxin (*seb*), and the protease genes (*splA*), and (*sspA*) [53]. Multiple halogenated indoles were tested against *S. aureus* to test for potential anti-virulence effects. The 5-iodoindole compound displayed greater potency compared to indole and could efficiently inhibit biofilm formation as well as carotenoid pigment production by *S. aureus* at 0.3 mM (1/10 of MIC) [54].

#### 2.2.2. Tetrangomycin Derivatives

Tetrangomycin was found to exhibit a significant cytotoxic effect on different cancerous cell lines as well as being a potent free radical scavenger [55]. Tetrangomycin derivatives have shown promising anti-virulence activity against *S. aureus* through STX biosynthesis inhibition. Ribeiro et al. conducted a study to test for the inhibitory characteristics of 27 tetrangomycin derivatives against *S. aureus* carotenoid pigment. The findings unveiled two of the most potent inhibitory compounds, naphthoquinone dehydro-α-lapachone and 2-Isopropylnaphtho[2,3-b] furan-4,9-dione, and revealed the importance of the presence of lipophilic and hydrogen acceptor moieties around the naphthoquinone ring to achieve STX inhibition. Spectrophotometric analysis was carried out as a simple means for examining the derivative’s mechanism of action on the STX biosynthetic pathway [56].

### 2.3. Repurposing FDA-Approved Drugs

#### 2.3.1. Cholesterol-Lowering Agents

In accordance with Oldfield, the catalysis of two farnesyl diphosphate (FPP) molecules into presqualene diphosphate by CrtM is considered the first basic step in the biosynthesis of staphyloxanthin by *S. aureus*. The resemblance in structure between CrtM and human squalene synthase (SQS), responsible for cholesterol biosynthesis in humans, aided the repurposing of some cholesterol-lowering agents into STX blockers (Figure 1). The utilization of cholesterol inhibitors as anti-virulence drugs has caused a significant inhibition to *S. aureus* virulence through directly inhibiting the carotenoid pigment production, rendering the treated strains vulnerable to the oxidative stress of human innate immunity and hence rapid clearance of the microorganism [57].

In 2008, a study conducted by Liu et al. suggested evident structural resemblance between *S. aureus* CrtM and human SQS. In attribution to the deduction, the analogy between the biosynthetic pathway of cholesterol in humans and that of STX production in *S. aureus* could be further studied. One cholesterol-lowering agent has been previously tested as a successful STX inhibitor that rendered *S. aureus* susceptible to oxidative stress of the human neutrophils in a mouse model after depigmenting the strain [16]. Later in 2009, Song et al. evaluated the possibility of inhibiting CrtM by potent phosphonosulfonates especially with halogen substitution and were able to prove their inhibitory effect on STX production with no effect against human squalene synthase [58].

Similarly, phosphonoacetamides were tested for STX inhibition in vitro and in a mouse infection model where a significant inhibition of disease progression was evident in the latter. X-ray crystallography revealed the most active compound to be *N*-3-(3-phenoxyphenyl) propylphosphonoacetamide [59].

**Figure 6:**
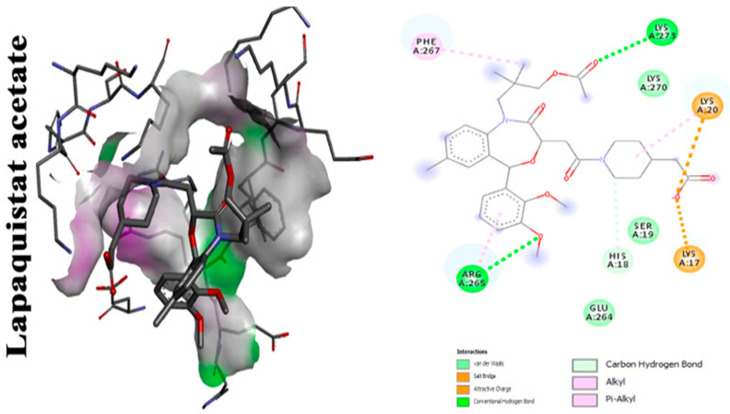
Molecular docking analysis showing 2D (on the **right** panel) and 3D (on the **left** panel) representation of interaction patterns of lapaquistat acetate with dehydrosqualene synthase receptor [25].

With focus on cholesterol-lowering drugs, lapaquistat acetate and squalestatins are reported to inhibit SQS in humans and hence their cholesterol-lowering activity. Molecular docking analysis was performed to detect the mode of binding of lapaquistat acetate and squalestatin analogs to CrtM enzyme of *S. aureus* (Figure 6). Molecular docking confirmed the involvement of specific target sites on the CrtM enzyme when introduced to the respective SQS inhibitors. Among the most prominent target residues were His18, Arg45, Asp48, Asp52, Tyr129, Gln165, Asn168 and Asp172 [60].

#### 2.3.2. Glyceryl Trinitrate (GTN)

GTN is a well-known medication for the treatment of cardiovascular diseases. Not only does GTN provide renowned anti-angina effects, but in a recent study it was also evident that this drug is capable of STX inhibition, biofilm disruption and oxidative stress resistance in *S. aureus* strains. Regarding in silico studies, it is reported that GTN binds with high affinity to CrtM which explains its marked STX inhibition activity. Thus, GTN could be a promising antipathogenic candidate against *S. aureus* [61].

#### 2.3.3. Diclofenac

Abbas et al. proposed that the renowned anti-inflammatory drug diclofenac possesses a notable anti-virulence effect against MRSA strains. The assumption was based on the discovery of the drug antipathogenic activity against *Pseudomonas aeruginosa* and *Proteus mirabilis.* In this work, diclofenac exerted an anti-STX production activity against MRSA clinical isolates at sub-MICs that reached 8–57.2% when compared to controls. Diclofenac treatment resulted also in decreased biofilm formation (22.67–70%) and noteworthy inhibition of hemolysin activity (5.4–66.34%). The phenotypic results were further confirmed by transcriptomic analysis using quantitative real time PCR that revealed marked downregulation of the previously tested virulence genes. Hence, diclofenac therapy along with other antimicrobials is recommended as an anti-virulence treatment against deleterious MRSA strains [62].

#### 2.3.4. Domperidone

Domperidone, an FDA-approved antiemetic drug, was studied by El-Ganiny et al. to detect its potential anti-virulence activity against *S. aureus*. Significant inhibition of the carotenoid pigment of *S. aureus* was detected using sub-inhibitory concentrations of domperidone to reach 76.4–81.23% at 1/8 MIC (9.8 μg/mL) and 1/4 MIC (19.5 μg/mL), respectively. Furthermore, the inhibition of the biofilm formation using sub-inhibitory concentrations of domperidone reached 84.37% at 1/4 MIC and 80.16% at 1/8 MIC. Gene expression analysis using qRT-PCR further confirmed the phenotypic results revealing decreased expression levels of virulence genes such as CrtM, SigB, SarA, AgrA, hla, fnbA, and icaA by domperidone treatment [63].

#### 2.3.5. Candesartan

Candesartan, a widely used drug in the treatment of high blood pressure, is now being re-studied for inherent anti-virulence characteristics against *S. aureus*. Candesartan’s ability to inhibit the antioxidant carotenoid pigment of *S. aureus* was evaluated using sub-inhibitory concentrations of the drug to yield pigment inhibition of 85.57% at 1/4 MIC (1.2 μg/mL) and 80.57% at 1/8 MIC (0.6 μg/mL), respectively. Furthermore, the inhibition of the biofilm formation using sub-inhibitory concentrations of domperidone reached 87.63% at 1/4 MIC and 71.5% at 1/8 MIC. Quantitative gene analysis revealed downregulation of virulence genes of *S. aureus* with the greatest inhibition activity against CrtM, sigB, sarA, agrA, hla and icaA genes [63].

#### 2.3.6. Antifungal Agents

Feifei et al. reported that the antifungal naftifine exerted a potent STX inhibitory activity via competitive inhibition of CrtN enzyme when tested on MSSA cells. The drug was capable of inhibiting the carotenoid pigment without affecting the growth of MSSA cells in a dose-dependent manner (up to 0.2 mM ~64.8 μg/mL) [64]. Later in 2020, Jing et al. proposed the synergistic role of naftifine to photodynamic antimicrobial chemotherapy (PACT) against *S. aureus*. The aiding role of naftifine is believed to be due to its inhibitory activity to STX that scavenges the reactive oxygen species (ROS) generated by PACT. Hence, the notorious antifungal resulted in an enhanced PACT activity when incubated with *S. aureus* cells at a concentration of 10 μM [65]. 

A recent study carried by El-Ganiny et al. focused on miconazole, which was reported to exhibit anti-virulence effects when studied against *S. aureus* standard strain (well-characterized strain with defined susceptibility or resistance profiles to the antimicrobial agents tested). In that study, 1/4 MIC (18.75 μg/mL) and 1/8 MIC (9.4 μg/mL) of miconazole were used to inhibit multiple virulence characteristics of *S. aureus*. STX inhibition reached 76.43–83.93% upon treatment with the indicated sub-inhibitory concentrations of the drug. Furthermore, the inhibition of the biofilm formation using sub-inhibitory concentrations of domperidone reached 90% at 1/4 MIC and 86.84% at 1/8 MIC. Transcriptomic analysis using qT-PCR divulged the reduced expression of CrtM, SigB, SarA, AgrA, hla, FnbA, and IcaA [63].

### 2.4. Newly Discovered CrtN Inhibitors

#### 2.4.1. 5 m Analog

Wang et al. have previously revealed that CrtN is a promising target for anti-virulence therapy. They have further disclosed the ability of the famous antifungal naftifine to drastically abolish the carotenoid pigment production in *S. aureus* species. The discovery of 5 m, a novel type of Benzofuran-derived CrtN inhibitor, has recently followed in the footsteps of the repurposing strategy of naftifine. As a result, the analogy has reflected a typical effect of the 5 m analog on *S. aureus* Newman and three other methicillin-resistant strains with low IC50 values ranging from 0.38–5.45 nM. The treated cells were rendered susceptible to immune clearance and their virulence was markedly weakened [66].

#### 2.4.2. Compound NP16

A newly discovered compound termed NP16 has showed a potent activity as a CrtN inhibitor in *S. aureus* strains. Consequently, notable interruption to the golden carotenoid pigment biosynthesis was evident that further caused an increased vulnerability to oxidative stress and neutrophil killing in vivo [67].

#### 2.4.3. 1,4-Benzodioxan-Derivatives

In 2018, 38 1,4-benzodioxan-derived CrtN inhibitors were synthesized to combat the downsides of the leading compound 4a. Derivative 47 exhibited a remarkable CrtN inhibitory effect with higher potency than 4a (pigment inhibition in *S. aureus* Newman: IC50 = 270.4 ± 43.8 nM by compound 47 vs. IC50 = 1.9 nM by compound 4a) in addition to enhanced water solubility. The sensitization effect of derivative 47 on MRSA strains was reported to be quite significant and successfully facilitated immune clearance in vitro [68].

### 2.5. Others

#### Farnesol

*Candida albicans* and *S. aureus* are among the most commonly known opportunistic pathogens that usually co-exist in mixed biofilms [69]. Both pathogens are often isolated together from hospital-admitted patients [70]. In a recent study, the role of *C. albicans*-secreted quorum sensing (QS) molecule (farnesol) was assessed against *S. aureus* cells. The study mimicked a mixed biofilm of *C. albicans* with *S. aureus* cells by repetitive exposure of *S. aureus* to farnesol. The sensitized *S. aureus* cells revealed significant inhibition of STX. The findings of transcriptional analysis further displayed marked changes in the expression of global regulators involved in resistance to oxidative stress. Unfortunately, the activation of stress-response mechanisms in *S. aureus* boosted its tolerance to intracellular killing and ROS. The pigment inhibition effect was reasoned then by proposing a theoretical binding model that indicated the binding of farnesol to CrtM enzyme causing blockage of the biosynthetic pathway of STX due to its structural resemblance to the substrate of CrtM. Those findings illustrate the role of the fungal-secreted QS mediator that could successfully elicit oxidative stress on *S. aureus* through thiol-based redox system activation. Moreover, the results of the previous study reported that depigmentation mediated by STX inhibitors was considered a transient conditional state, as upon the gradual removal of farnesol, gradual recovery of the pigment was observed in comparison with the control cells [71].

**Table 1:** Chemical structures of various staphyloxanthin inhibitors.

STX Inhibitors	Chemical Structure
Flavone	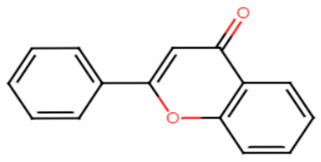
2. Myricetin	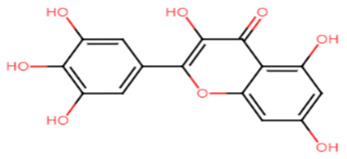
3. Rhodomyrtone	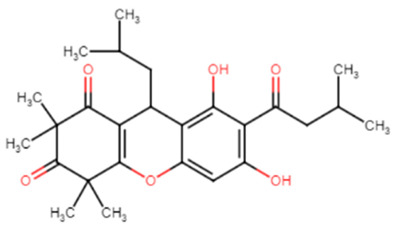
4. Chitosan	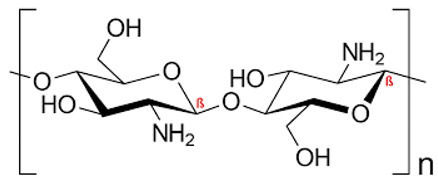
5. E-nerolidol (derived from *P. heyneanus* and *C. tamala* essential oils)	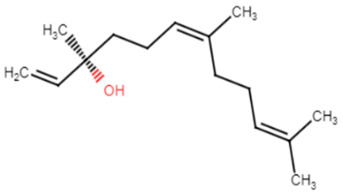
6. 2-hydroxy-4-methoxybenzaldehyde (HMB) [30]	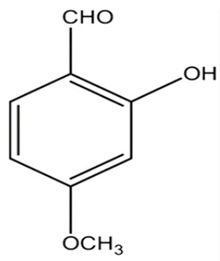
7. Myrtenol	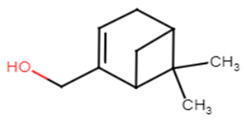
8. Euphol (derived from *Euphorbia tirucalli* latex) [72]	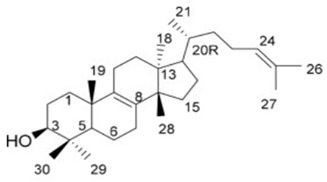
9. *cis*-β-terpineol (derived from *Schinus terebinthifolia* leaf lectin) [37]	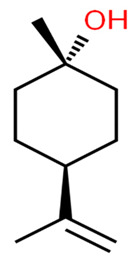
10. Pulverulentone A (C1) (derived from *Callistemon citrinus* Skeels) [40]	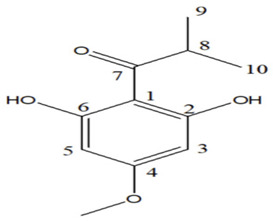
11. δ-cadinene (derived from the essential oil of *Eugenia brejoensis L.*) [41]	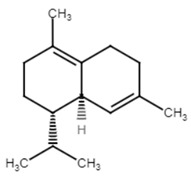
12. Ginkgoic acid (derived from *Ginkgo biloba* exocarp extract) [73]	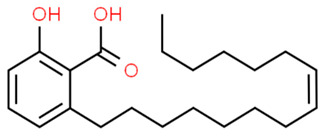
13. Carvacrol	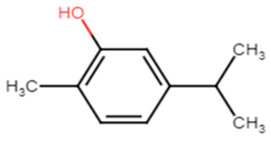
14. Thymol	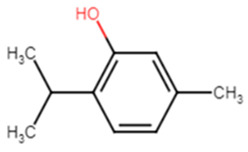
15. Hesperidin	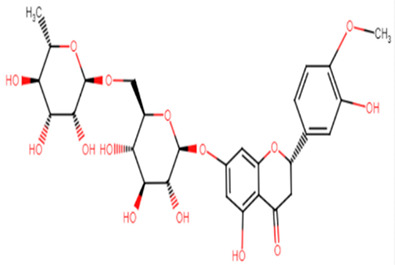
16. 7-benzyloxyindole	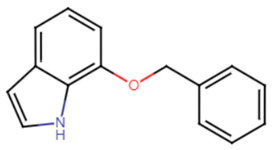
17. Tetrangomycin	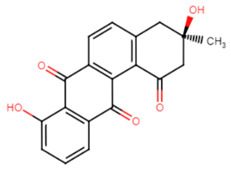
18. 2-isopropyl-naphtho[2,3-b] furan-4,9-dione (tetrangomycin derivative)	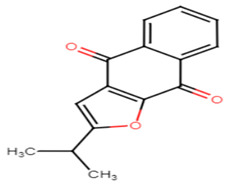
19. Lapaquistat acetate	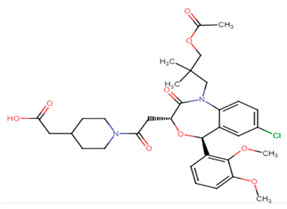
20. Squalestatin	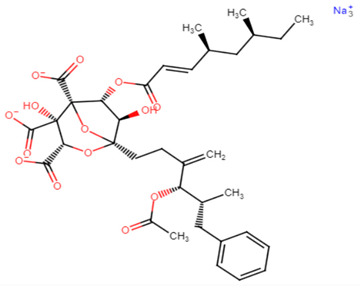
21. Glyceryl trinitrate	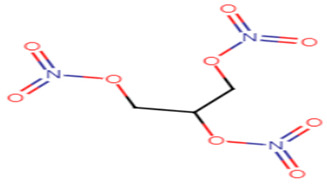
22. Diclofenac sodium	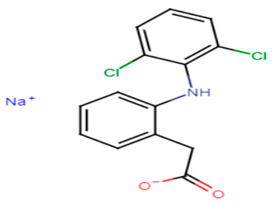
23. Domperidone	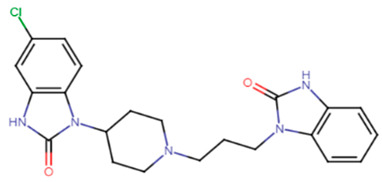
24. Candesartan	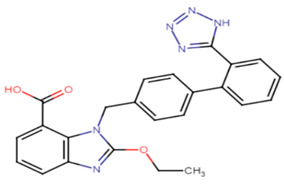
25. Naftifine	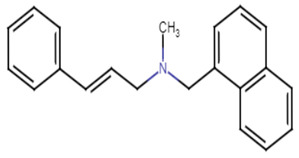
26. Miconazole	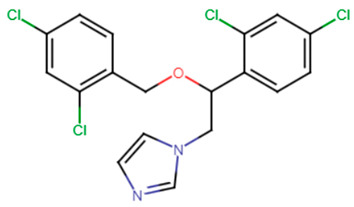
27. 5M analog [66]	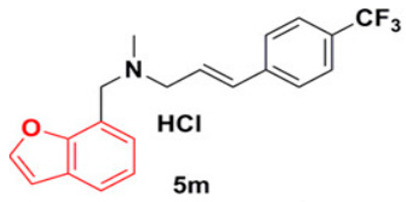
28. NP16 [67]	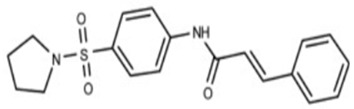
29. Derivative 47 [68] 4-Benzodioxine-7-methyl)-N-methyl-5-(4-trifluorophenyl)prop-2,4-dien-1-amine Hydrochloride	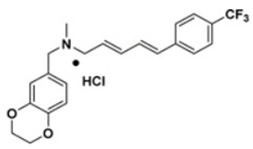
30. Farnesol	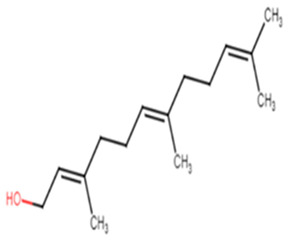

Chemical structures are from Reaxys website via Egyptian Knowledge Bank (EKB) 2022 (https://081291uul-1104-y-https-www-reaxys-com.mplbci.ekb.eg/) (accessed on 14 February 2022).

**Table 2:** Structure activity relationship (SAR) of previously studied STX inhibitors.

Compound	Structure Activity Relationship (SAR)
Flavone	Carbonyl moiety is crucial for activity, interacts with adjacent amino acid residues in CrtM receptor by conventional hydrogen bond. Yet, the exact mechanism of action for anti-virulence activity remains to be determined. [10,20,74]
Myricetin	Hydroxyl moiety enhances the binding affinity to adjacent amino acid residues of the CrtM receptor through conventional hydrogen bonds. Carbonyl group is essential for activity, binds to adjacent amino acids of the receptor via hydrogen bonds [21,74].
Rhodomyrtone	Alkyl interactions with CrtM receptor at CH3 terminals. Carbonyl moiety is crucial for activity, interacts with VAL in CrtM receptor by hydrogen bond. Hydroxyl moiety enhances the binding affinity to LYS residues through conventional hydrogen bonds [24,25].
E-nerolidol	The alcoholic moiety is essential for activity, binds to CrtM through hydrogen bond. The backbone of the structure interacts with the hydrophobic pocket of the receptor [28].
Carvacrol	Essential phenolic hydroxyl group for activity, binds with conventional hydrogen bond to CrtM. Oxygen involved in the hydroxyl group interacts with GLY A:161 through carbon hydrogen bond. Phenyl ring binds by Pi–Pi T-shaped bond to PHE A:22. Terminal methyl groups bind to ALA A:157, ILE A:241 and PHE A:22 [25].
Thymol	Essential phenolic hydroxyl group for activity, binds with conventional hydrogen bond to CrtM. Oxygen involved in the hydroxyl group interacts with PHE A:117 through carbon hydrogen bond. Methyl terminals bind to TYR A:129, LYS A:113 and PHE A:120 through alkyl interactions [49].
Hesperidin	In case of CrtM, hesperidin actively interacts through (Arg 158, Tyr 154 and Gln 102). Carbonyl moiety interacts with amino acid residues via hydrogen bond. Hydroxyl groups enhances the activity [51].
Tetrangomycin	Hydrogen acceptor groups are crucial for activity. Lipophilic moieties decorating the naphthoquinone ring enhance STX inhibition [55,56].
7-benzyloxyindole	The presence of ether group (acidic moiety) enhances the activity of the compound. The addition of a second hydrophobic ring enhances the activity [53,75].
2-isopropylnaphtho [2,3-b]furan-4,9-dione	Hydrogen acceptor groups are crucial for activity. Lipophilic moieties decorating the naphthoquinone ring enhance STX inhibition [55,56].
Lapaquistat acetate	Carbonyl groups interact with adjacent amino acid residues in CrtM receptor via conventional hydrogen bonds. Aromatic ring interaction with adjacent amino acid residues via alkyl interactions. Methyl group interacts with PHE A:267 on the receptor via Pi–alkyl interaction [25].
Squalestatin	Interaction of carbonyl groups, hydroxyl groups and aromatic benzene ring with His18, Arg45, Asp48, Asp52, Tyr129, Gln165, Asn168 and Asp172 residues on CrtM receptor [60].
Glyceryl trinitrate	GTN has nine hydrogen bonds with Arg45, Tyr129, Gln165, Asn168, Val 133 and Tyr248 electrostatic interaction with Arg45 and Asp48 and pi-cation interaction of the nitrogen atom with Tyr183 [61].
Naftifine	The naphthalenyl moiety of NTF is not indispensable for pigment inhibitory activity, the *N*-methyl group is critical for high potency on CrtN receptor [66].
5M analog	The naphthalenyl moiety of NTF is not indispensable for pigment inhibitory activity, the *N*-methyl group is critical for high potency, the 4-substituted phenyl moiety is critical for high potency on CrtN receptor. The para-position is the best substituted position at the phenyl ring [66].
Derivative 47	The *N*-methyl group is critical for high potency. Unsubstituted alkenyl linker was critical for improving pigment inhibitory activity. The 4-substituted phenyl moiety is critical for high potency on CrtN receptor. The para-position is the best substituted position at the phenyl ring [66,68].

## 3. Naturally Existing Susceptible Variant

In 2013, the hypothesis of linking virulence attenuation to pigment inhibition was further investigated. Clonal complex 75 of *S. aureus* (*S. argenteus*) revealed a lack of STX regulating operon, CrtOPQMN, which divulged its inability to produce the carotenoid pigment and hence its vulnerability to ROS and neutrophil clearance when experimented on in vitro as well as in vivo. On the other hand, deliberately transformed cells with pTX crtOPQMN lead to increased resistance to oxidative stress [76].

## 4. Reversing STX Inhibition

### 4.1. Inter-Species Communication

Quorum sensing (QS) or cell–cell communication is a pivotal process governed by some secreted mediators that allow the recognition of one species to the other and behavioral coordination in accordance. Hence, the secreted molecules can remarkably alter the cell physiology of the species present in a polymicrobial domain [77]. *S. aureus* strains are naturally present as yellow and white colony variants. It is believed that bacterial pigments are usually synthesized in stressful conditions.

Owing to the fact that quorum sensing can control pigment production in a variety of bacterial cells, as discussed earlier, coinfection with *C. albicans* resulted in inhibition of *S. aureus* STX pigment due to the secretion of farnesol. However, co-infection with *Pseudomonas aeruginosa* resulted in increased production of STX and catalase in the white variants of *S. aureus* through QS. The induced carotenoid pigment by *P. aeruginosa* conferred polymyxin resistance and hydrogen peroxide endurance upon the respective *S. aureus* cells. Thus, it is assumed that the STX biosynthesis mechanism is always present in *S. aureus* variants which can be induced by the chemical signaling molecules of *P. aeruginosa* and inter-species interaction [78].

### 4.2. Inactivation of Catabolite Control Protein E (CcpE)

Metabolism in *S. aureus* is crucially regulated by the catabolite control protein E (CcpE) where catalase binds to stimulate the expression of almost 4.7% of the *S. aureus* genetic material. Surprisingly, it was found that the inactivation of CcpE has led to enhancing *S. aureus* virulence to include increased STX production, resistance to whole-blood killing and iron scavenging. CcpE is now known as *S. aureus* metabolic sensor that allows the strains to regulate virulence expression [79].

## 5. Genetic Manipulation

### 5.1. msaABCR Operon

*S. aureus* has developed an arsenal of defense mechanisms mediated by a complex grid of regulators that allows the strains to survive under oxidative stress. According to Pandey et al., *msaABCR* operon was depicted to be a stress regulator in *S. aureus* cells which conveyed the character of resistance to antibiotics and oxidative stress and aided the generation of dormant cells of *S. aureus* that in turn exhibited multidrug tolerance. Transcriptional analysis revealed downregulation of multiple genes responsible for resistance against oxidative stress in response to the deletion of *msaABCR* operon. Genes involved in STX as well as the ohr gene (responsible for the cellular defence against organic hydroperoxides) were also downregulated upon the deletion of the operon. Moreover, it has been reported that MsaB, a protein product of the *msaABCR* operon, can regulate the expression of crtOPQMN operon (responsible for the production of the golden pigment) and the ohr gene as well as its repressor, resulting in a controlled resistance to oxidative stress in vitro. This study casts a light on the crucial role of the *msaABCR* operon and its protein product MsaB in defending *S. aureus* against unfavorable environmental conditions as oxidative stress. Based on this discovery, the operon could be a prospective target for antipathogenic treatment against the recurrent staphylococcal infections [80].

### 5.2. SigB

The expression of the golden yellow pigment is governed by the global stress response regulator SigB. The presence of SigB allows *S. aureus* to increase the expression of multiple resistance genes including the ones responsible for oxidative stress resistance manifested in the eponymous STX pigment. In a recent study, radiation survival of *S. aureus* was examined to detect the protective role of SigB against radiation damage. Mutant strain cells of *S. aureus* lacking the carotenoid pigment (crt enzymes-deficient) were subjected to different types of radiation. The crt-mutant cells were reported to be threefold more susceptible to UV radiation than the wild-type strain [81].

## 6. Possibility of an Emerging Resistance to Anti-virulence Therapy

The target of anti-virulence agents is to disrupt the pathogen’s virulence without affecting its growth or viability, thus preserving the normal microbiota, avoiding selective pressure on the bacteria and consequently reducing the emergence of antimicrobial resistance. However, several in vitro studies observed that there could be an emerging resistance to certain anti-virulence agents, but not all agents have the same risk of resistance [82,83,84]. Anti-virulence agents targeting a specific mechanism of virulence may have a limited probability of resistance compared to other agents targeting regulatory genes that may greatly affect multiple virulence factors. The emergence of resistance could be attributed to selective pressure on microbial subpopulation; however it is almost very weak in comparison with conventional antibiotics [85].

## 7. Conclusions

In this review we have discussed several naturally occurring as well as chemically synthesized STX inhibitors, hence avoiding selective pressure on *S. aureus* strains as a new approach to combat bacterial infections without drug resistance development. The significance of QS in regards to upregulation and downregulation of STX biosynthesis has been confirmed. Anti-virulence therapy is a novel approach towards a bacterial resistance-free regimen for a variety of infections. This approach is currently under preclinical investigations and it is recommended to proceed to clinical trials on human models after succeeding with in vitro and in vivo mouse models as well as invertebrate models. The STX inhibitors are considered potential anti-*S. aureus* agents particularly for the control of MRSA, a life-threatening pathogen of clinically relevant importance.
